# The Aurora kinase A inhibitor TC-A2317 disrupts mitotic progression and inhibits cancer cell proliferation

**DOI:** 10.18632/oncotarget.12448

**Published:** 2016-10-04

**Authors:** Yoo Hong Min, Wootae Kim, Ja-Eun Kim

**Affiliations:** ^1^ Department of Biomedical Science, Graduate School, Kyung Hee University, Seoul 130-701, Republic of Korea; ^2^ Department of Pharmacology, School of Medicine, Kyung Hee University, Seoul 130-701, Republic of Korea

**Keywords:** Aurora kinase A, TC-A2317, mitosis, spindle assembly checkpoint

## Abstract

Mitotic progression is crucial for the maintenance of chromosomal stability. A proper progression is ensured by the activities of multiple kinases. One of these enzymes, the serine/threonine kinase Aurora A, is required for proper mitosis through the regulation of centrosome and spindle assembly. In this study, we functionally characterized a newly developed Aurora kinase A inhibitor, TC-A2317. In human lung cancer cells, TC-A2317 slowed proliferation by causing aberrant formation of centrosome and microtubule spindles and prolonging the duration of mitosis. Abnormal mitotic progression led to accumulation of cells containing micronuclei or multinuclei. Furthermore, TC-A2317–treated cells underwent apoptosis, autophagy or senescence depending on cell type. In addition, TC-A2317 inactivated the spindle assembly checkpoint triggered by paclitaxel, thereby exacerbating mitotic catastrophe. Consistent with this, the expression level of Aurora A in tumors was inversely correlated with survival in lung cancer patients. Collectively, these data suggest that inhibition of Aurora kinase A using TC-A2317 is a promising target for anti-cancer therapeutics.

## INTRODUCTION

Mitosis is a critical step in the segregation of chromosomes to daughter cells. Proper chromosome segregation is ensured by the spindle assembly checkpoint (SAC), also known as the mitotic checkpoint, which is regulated by a diverse set of kinases [1]. Among the factors responsible for the proper progression of mitosis are the Aurora family of serine/threonine kinases [2]. The first Aurora kinase, discovered in a *Drosophila melanogaster* mutant with monopolar spindles due to defect in centrosome seperation, is functionally related to Increase-in-ploidy 1 (IPL1) in *Saccharomyces cerevisiae*, Aurora-related kinase 1 (ARK1) in *Schizosaccharomyces pombe*, and Aurora/IPL1-related kinase (AIK) in mammals [3]. The three mammalian Aurora paralogs are Aurora A, Aurora B and Aurora C. Aurora C primarily regulates the meiotic cell cycle, but its function remains incompletely understood [4]. Aurora B regulates chromosome condensation, chromosome biorientiation, SAC, regulation of sister chromatid cohesion, spindle disassembly, and cytokinesis [5].

Aurora A is also known as serine/threonine protein kinase 15 (STK15), serine/threonine protein kinase 6 (STK6), breast tumor-amplified kinase (BTAK), Aurora-related kinase 1 (ARK-1), Aurora/IPL1-related kinase (AIK), and Aurora 2. It controls mitotic regulatory functions in centrosome maturation, centrosome separation, mitotic entry, chromosome alignment, bipolar spindle formation, and cytokinesis; consequently, tight regulation of Aurora kinase A is required for proper mitotic progression [6, 7]. Depletion of Aurora A induces monopolar spindle formation, prometaphase arrest, aneuploidy, apoptosis, or senescence [8]. Overexpression of Aurora A induces centrosome amplification, multipolar spindle formation, aneuploidy, and defects in the SAC [9, 10]. Recently, Aurora A was proposed to play non-mitotic regulatory functions in cell migration, cilium disassembly, calcium signaling, cell polarity, DNA repair, DNA replication, pluripotency, p53 activity, and NF-κB signaling [6, 8].

The various functions of Aurora A are governed by localization, expression, and activity. Aurora A is localized to the centrosome from late S to early G1 phase, and is also localized in the spindle pole and spindle microtubules throughout mitosis, consistent with its roles in the centrosome and spindle [11]. Expression of Aurora A protein is low in G1/S phase, but peaks at G2 and early mitosis [10]. Over the course of the cell cycle, its expression is regulated at the transcriptional level; specifically, it is positively regulated by E4TF1 and repressed by tandem elements, the CDE (cell cycle-dependent element) and CHR (cell cycle gene homology region) sequences [12]. Upregulated Aurora A protein is degraded in late mitosis following recognition of its N-terminal A-box and C-terminal D-box by the Cdh1-dependent anaphase-promoting complex/cyclosome (APC/C) although the N-terminal KEN-box is not required at this stage [6]. Degradation is completed in G1 phase. In addition to the APC/C, several other factors including Chfr, Fbxw7, AURKAIP1, Az1, GSK-3β, and PP2A regulate Aurora A protein expression [6]. The activity of Aurora kinase A is increased by autophosphorylation of T288 in the activating T-loop. This autophosphorylation is activated by cofactors such as TPX2, Ajuba, NEDD9, Bora, calmodulin, and PAK1 at centrosome; T288 is dephosphorylated by PP1 [6].

The *AURKA* gene on chromosome 20q13 is amplified, or Aurora A is overexpressed, in a wide range of cancers including bladder, breast, colorectal, gastric, head and neck, liver, lung, neuronal, ovarian, and prostate cancer, leukemia and lymphoma [8]. This amplification/overexpression is associated with unfavorable prognosis and low survival. Aurora A overexpression induces cell transformation [13] and mammary tumor development [14]. Aurora B is also overexpressed in many types of cancers, but its role in tumorigenesis has not been clearly defined [15]. Therefore, specific inhibition of Aurora kinase A may be useful as a cancer treatment. Several specific Aurora kinase A inhibitors, including ENMD-2076, MK-5108 (VX-689), MLN-8054, and MLN-8237 (alisertib), are undergoing clinical trials [8, 16, 17]. Although TC-A2317 was developed as a specific Aurora kinase A inhibitor [18], its anti-tumor effect has been investigated only in glioblastoma [19], and its mechanism has not been elucidated. In this study, we found that TC-A2317 also inhibits lung cancer cell proliferation by inducing mitotic catastrophe, suggesting that it might be effective against lung cancer.

## RESULTS

### TC-A2317 decreases cell survival

**Figure 1:**
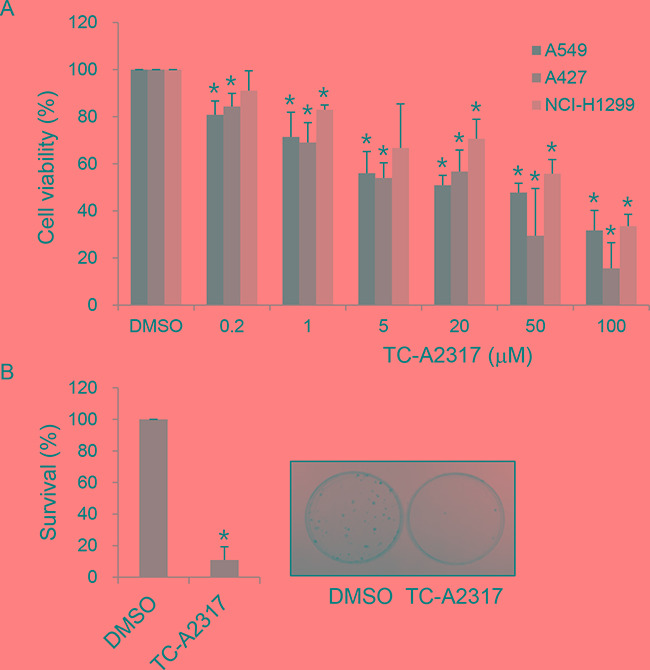
TC-A2317 inhibits cell proliferation **A.** A549, A427 and NCI-H1299 cells were treated with various concentrations of TC-A2317 for 24 hr. Cell viability was determined using the MTT assay. **B.** A549 cells were treated with 1 μM TC-A2317 for 24 hr. After removal of TC-A2317, the cells were seeded for colony growth. Colonies were counted after 14 days. All values from three independent experiments are represented as means ± standard deviation (n=3). Asterisks (*) represent statistically significant differences (*P* < 0.05, Student's *t*-test).

We aimed to determine the short- and long-term effect of pharmacological inhibition of Aurora kinase A activity on the survival of lung cancer cells. For this purpose, we treated A549, A427 and NCI-H1299 cells with TC-A2317, a specific Aurora kinase A inhibitor. Treatment of cells with TC-A2317 for 24 hr significantly decreased cell viability in a dose-dependent manner (Figure 1A). In addition, A549 cells treated with TC-A2317 showed dramatically reduced colony-forming activity, indicating that the drug exerted a long-term effect (Figure 1B). Together, these results show that TC-A2317 decreases the survival of lung cancer cells.

### TC-A2317 causes cells to undergo abnormal cell division

**Figure 2:**
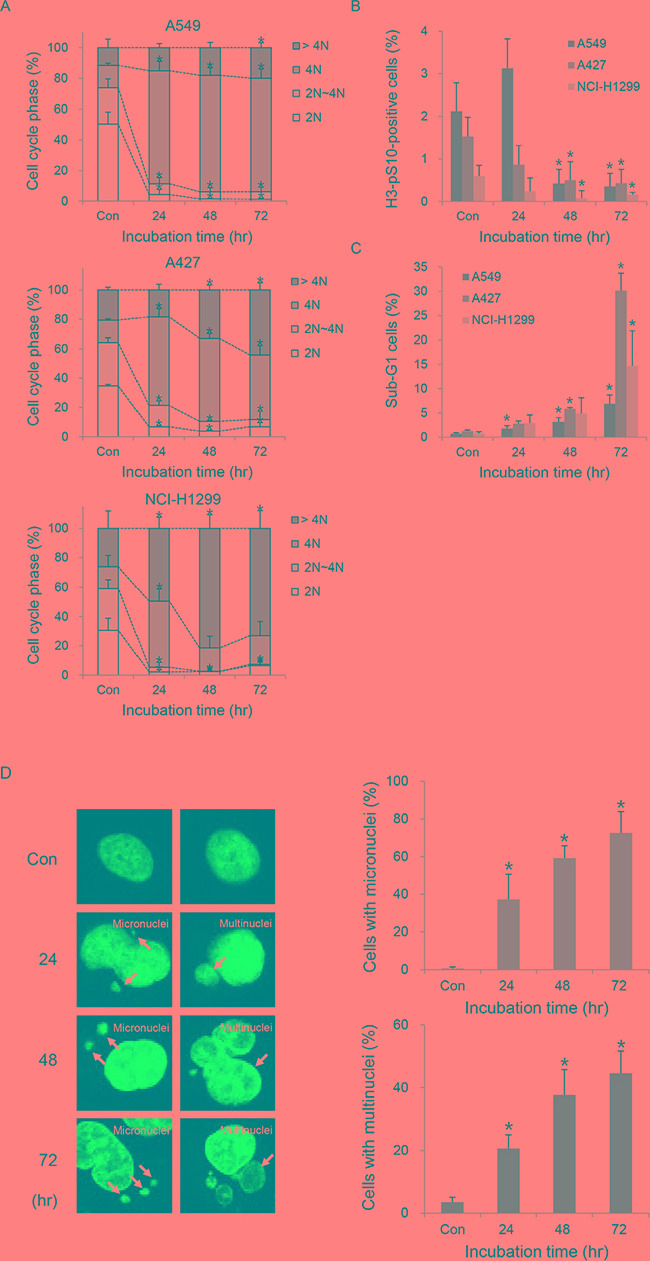
TC-A2317 induces the abnormal progression of cell cycle **A-C.** A549, A427 and NCI-H1299 cells were treated with 1 μM TC-A2317 for the indicated times. Cell cycle distribution (A), mitotic fraction (B), and sub-G1 population (C) were analyzed by flow cytometry by staining cells with PI and anti–H3-pS10. **D.** A549 cells were treated with 1 μM TC-A2317 for the indicated times. Nuclei were visualized by staining with Hoechst. Cells with micronuclei and multinuclei were counted. Con (Control) means cells treated with vehicle (DMSO) for 72 hr. All values from three independent experiments are represented as means ± standard deviation (n = 3). Asterisks (*) represent statistically significant differences (*P* < 0.05, Student's *t*-test).

We next investigated the causes of reduced cell survival following TC-A2317 treatment. Analysis of cell cycle distribution revealed that TC-A2317 treatment led to significant accumulation of cells with 4N DNA content in A549, A427 and NCI-H1299 cells (Figure 2A and Supplementary Figure S1A). To determine whether these cells represented a G2/M-arrested population, we monitored the level of H3-pS10, which is normally upregulated at late G2 and M phase. TC-A2317–treated cells exhibited a dramatic reduction in H3-pS10 level (Figure 2B and Supplementary Figure S1A), suggesting that the accumulation of cells with 4N DNA was not due to G2/M arrest. In addition, cells with >4N DNA content were significantly accumulated, indicating that TC-A2317 induces formation of polyploidy (Figure 2A and Supplementary Figure S1A). In particular, because NCI-H1299 cells are rapidly dividing, they showed even 16N DNA content (Supplementary Figure S1A). It demonstrates that all three cells are endoreduplicated. Simultaneously, sub-G1 population increased in TC-A-2317-treated cells in a time-dependent manner, implying that TC-A2317 induces cell death (Figure 2C and Supplementary Figure S1A). The most well-known Aurora A inhibitor, alisertib also results in the similar change of cell cycle in A549 cells (Supplementary Figure 1B). However, vehicle does not affect cell cycle phase, H3-pS10 level, and sub-G1 population (Supplementary Figure S1C). Nuclear staining also revealed that TC-A2317–treated A549 cells exhibited abnormal nucleation (Figure 2D). The proportions of cells exhibiting micronucleation and multinucleation increased significantly over time. Alisertib-treated cells also contained micronuclei and multinuclei (Supplementary Figure S2A). Together, these data demonstrate that Aurora A inhibition induces abnormal nuclear cell division and the formation of aneuploidy.

### TC-A2317 causes aberrant mitotic progression

**Figure 3:**
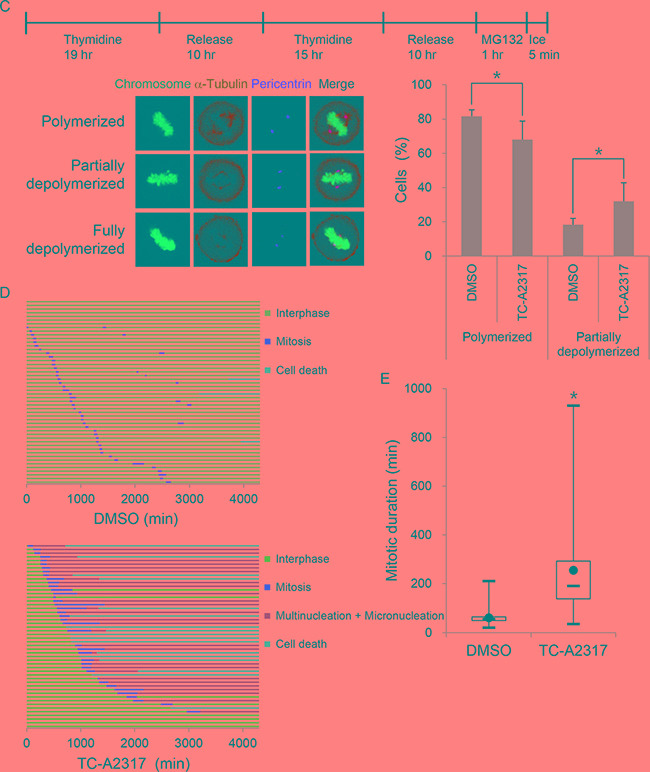
TC-A2317 results in abnormal centrosome formation, microtubule destabilization and prolonged mitosis **A-B.** A549 cells were treated with 1 μM TC-A2317 for the indicated times, and then subjected to immunofluorescence staining with antibodies against α-tubulin and pericentrin. Con (Control) means cells treated with vehicle (DMSO) for 24 hr. **C.** A549 cells were enriched in mitosis after release from a double-thymidine block, treated with 20 μM MG132 for 1 hr, and then treated with 1 μM TC-A2317 for 5 min on ice. Depolymerization of mitotic spindles was determined by visualizing mitotic spindles with α-tubulin antibody. The number of metaphase cells with depolymerized mitotic spindles was counted. All values from three independent experiments are represented as means ± standard deviation (n = 3). **D-E.** H2B-RFP–transduced A549 cells were treated with 0.1% DMSO or 1 μM TC-A2317 for 72 hr. Cells were monitored by time-lapse fluorescence microscopy. (D) Cells undergoing interphase, mitosis, multi- and micro-nucleation, and cell death were counted (n = 50). (E) Duration of mitosis was represented in box-and-whisker plot. Box, interquartile range; whisker, min and max; black circle, average; bar, median. Asterisks (*) represent statistically significant differences (*P* < 0.05, Student's *t*-test).

Aneuploidy, including micronucleation and multinucleation, can occur as a consequence of abnormal mitotic division. To determine whether asymmetrical centrosomes or abnormal mitotic spindles were responsible for the aneuploidy described above, we stained cells for pericentrin, a component of the pericentriolar material (PCM), and α-tubulin, respectively. A549 cells treated with DMSO normally displayed either one or two centrosome(s). A majority of interphase cells treated with TC-A2317 contained one centrosome, but the very small number of interphase cells contained two centrosomes, which are very adjacent (Figure 3A). However, alisertib-treated cells contained multiple centrosomes, which are closely located, as well as one centrosome (Supplementary Figure S2A). These observations suggest that Aurora A inhibition induced asymmetrical mitotic division. Because TC-A2317 reduced a proportion of mitotic cells and might induce cell cycle arrest in interphase after treatment for 24–72 hr (Figure 2B), we could not observe centrosomes and mitotic spindles during mitosis. Therefore, those were examined following a brief incubation with TC-A2317 for 30 min or 1 hr. First, while two centrosomes were separated toward opposing sides in control cells, they were not fully separated in TC-A2317-treated cells at prometaphase (Figure 3B and 6A, and Supplementary Figure S5A). It indicates that Aurora A inhibition leads to a failure in centrosome separation. Second, we determined the stability of mitotic spindles by performing microtubule depolymerization assays. The rate of microtubule depolymerization was higher in TC-A2317–treated cells than in controls cells (Figure 3C), indicating that TC-A2317 treatment destabilizes mitotic spindles. Overall, these results suggest that abnormal centrosomes and unstable mitotic spindles exacerbate mitotic catastrophe. Consistent with this, live-cell imaging revealed that TC-A2317 treatment resulted in formation of multi- and micro-nucleated cells and cell death, whereas control cells underwent division into two daughter cells (Figure 3D). In addition, the duration of mitosis was extended in TC-A2317–treated cells relative to control cells (Figure 3E); mitotic delay can result in cell death (Figure 1). Together, these data demonstrate that TC-A2317 treatment perturbs mitotic progression and contributes to the accumulation of chromosomal abnormalities.

### TC-A2317–treated cells undergo apoptosis, autophagy, or senescence

**Figure 4:**
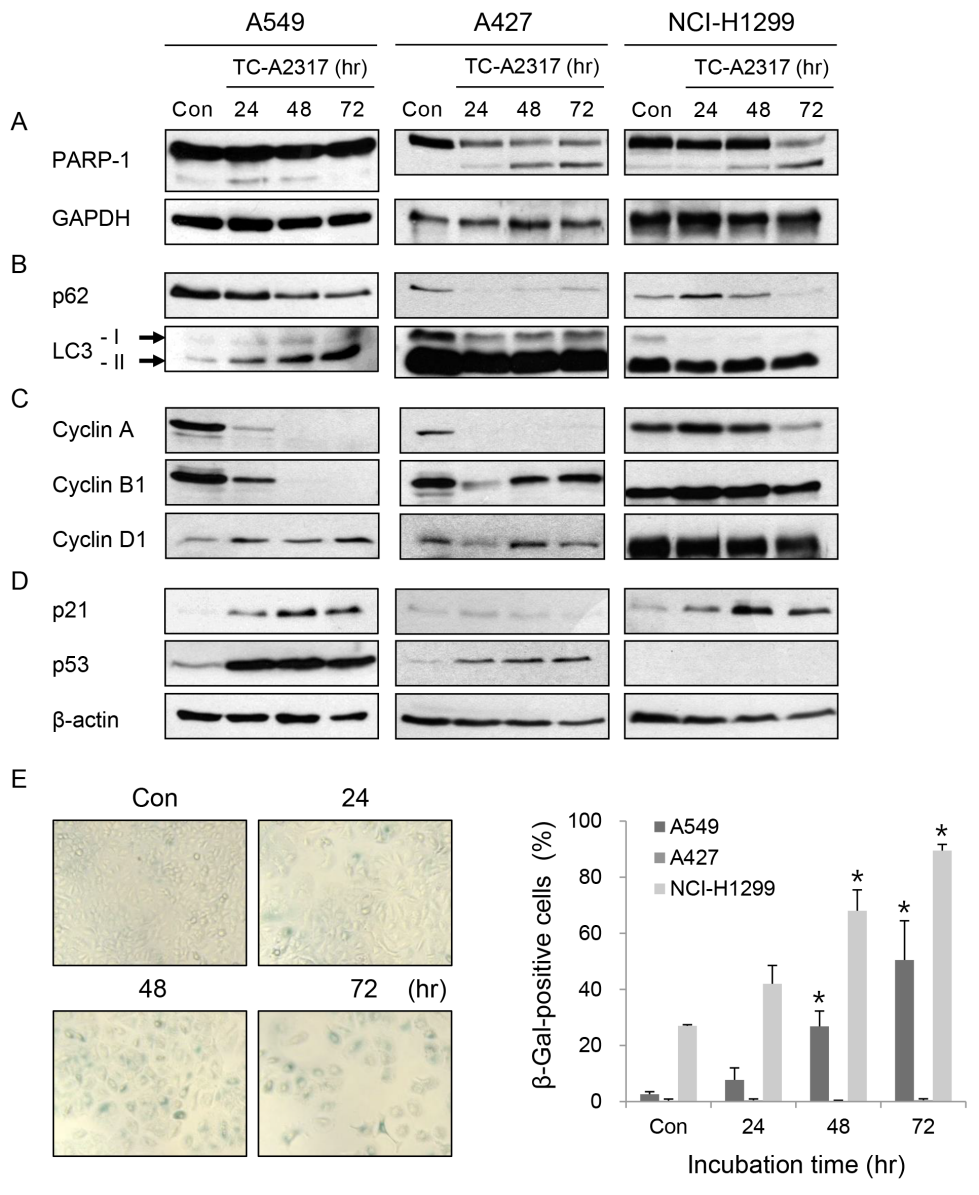
TC-A2317 induces apoptosis, autophagy or senescence **A-E.** A549, A427 and NCI-H1299 cells were treated with 1 μM TC-A2317 for the indicated times. Con (Control) means cells treated with vehicle (DMSO) for 72 hr. (A–D) Expression level of each protein was evaluated by Western blotting. (E) Senescence-associated β-galactosidase (SA-β-Gal) assay was performed and then SA-β-gal-positive cells out of total 250 cells were detected by staining. All values from three independent experiments are represented as means ± standard deviation (n = 3). Asterisks (*) represent statistically significant differences (*P* < 0.05, Student's *t*-test).

Next, we investigated the consequences of abnormal mitotic progression. First, we sought to explain the cell death observed in Figure 3D. In A549 cells, cleavage of PARP-1 was very slightly induced 24 hr after TC-A2317 treatment, but reduced at later stages of TC-A2317 treatment (i.e., at 48 and 72 hr; Figure 4A). It indicated that a minority of A549 cells had undergone apoptosis (Figure 4A). Thus, a subset of A549 cells overcame apoptosis and followed another fate. By contrast, cleavage of PARP-1 significantly increased in a time-dependent manner when TC-A2317 was treated in A427 and NCI-H1299 cells, indicating that they underwent apoptosis (Figure 4A). It implicates that lung cancer cells with different genetic background underwent different types of cell death. Another possible outcome was autophagy, reflected by reduced levels of p62/SQSTM1, an ubiquitin-binding scaffold protein, and elevated levels of LC3-II, a marker of autophagosome formation. TC-A2317 treatment enhanced autophagy-related changes in A549, A427 and NCI-H1299 cells (Figure 4B). Autophagy induced by TC-A2317 treatment could be causally related to low cell survival (Figure 1). In addition, the scarcity of mitotic cells (Figure 2B) suggested that TC-A2317 arrested cells at interphase. To characterize the cell cycle arrest in more detail, we determined the levels of cyclin A, cyclin B1 and cyclin D1, which are upregulated in G2, mitosis and G1, respectively. As expected from the data shown in Figure 2B, the levels of cyclin A and cyclin B1 were significantly reduced in TC-A2317–treated A549 and A427 cells, indicating that the cells were not arrested at G2 or mitosis (Figure 4C). Instead, the upregulation of cyclin D, suggesting a G1 arrest, was observed in A549, not in A427 cells (Figure 4C). However, the levels of cyclins were not dramatically changed in NCI-H1299 cells probably because they are rapidly dividing. Instead, to elucidate the mechanism by which TC-A2317 induces cell cycle arrest, we monitored the levels of p21, a cyclin-dependent kinase inhibitor, and its upstream transcription factor p53. Both p21 and p53 were highly upregulated in TC-A2317–treated A549 cells (Figure 4D). In A427 cells, only p53, not p21, was upregulated. By contrast, although NCI-H1299 cells are p53-null, p21 was upregulated after TC-A2317 treatment. Overall, it suggests that A549 and NCI-H1299 cells underwent p21-dependent cell cycle arrest, but A427 cells underwent p53-depenent apoptosis. To further verify that A549 and NCI-H1299 cells expressing high levels of p21 were senescent, we performed senescence-associated β-galactosidase (SA-β-gal) staining. SA-β-gal activity increased in TC-A2317–treated A549 and NCI-H1299 cells in a time-dependent manner, indicating that TC-A2317 induces the irreversible G1 arrest known as senescence (Figure 4E and Supplementary Figure S3B). As expected in Figure 4D, A427 cells did not undergo senescence (Figure 4E and Supplementary Figure S3B). It indicates that p21 upregulation is required for TC-A2317-induced senescence. The vehicle does not affect apoptosis, autophagy and senescence (Supplementary Figure S3A and S3B). In conclusion, major types of cell fate are autophagy and senescence in A549, apoptosis and autophagy in A427, and apoptosis, autophagy and senescence in NCI-H1299 cells, respectively. Overall, these data show that TC-A2317–induced chromosomal instability triggered apoptosis, autophagy or senescence although a type of cell death was dependent on cell type.

### TC-A2317–treated cells overcome paclitaxel-induced mitotic arrest

**Figure 5:**
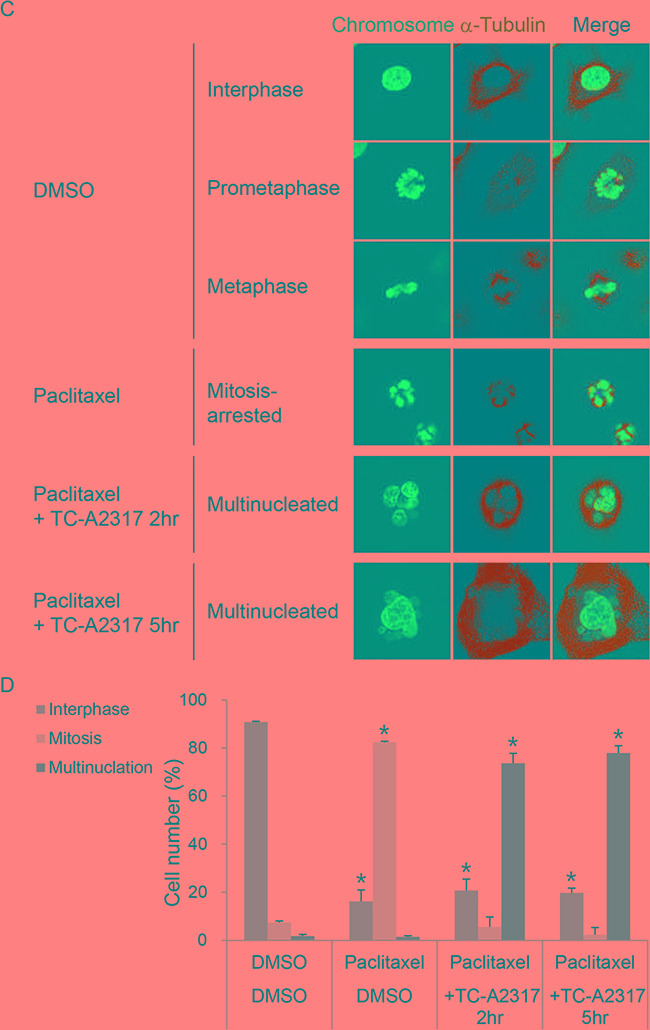
TC-A2317 promotes the exit from mitotic arrest A549 cells were pre-treated with 50 nM paclitaxel for 16 hr, and then treated with 0.5 μM TC-A2317 for the indicated times. **A.** DNA content and H3-pS10 level were determined by flow cytometry. **B.** H2B-RFP–transduced A549 cells treated with paclitaxel were monitored by time-lapse fluorescence microscopy immediately after addition of DMSO or TC-A2317 for 5 hr. Cells undergoing mitotic arrest, multinucleation, interphase, and cell death were counted (n = 44). **C.** The nucleus of the cells and the cell boundary were visualized by staining with Hoechst and α-tubulin, respectively. **D.** Multinucleated cells were counted. All values from three independent experiments are represented as means ± standard deviation (n = 3). Asterisks (*) represent statistically significant differences (*P* < 0.05, Student's *t*-test).

Next, we used mitosis-arrested cells to investigate the regulatory mechanism by which TC-A2317 affects abnormal mitotic progression. For this purpose, we induced mitotic arrest with paclitaxel, a microtubule stabilizer that interferes with normal microtubule dynamics and ultimately prevents attachment of mitotic spindles to kinetochores. The level of H3-pS10 increased dramatically in paclitaxel–treated cells, but decreased in a time-dependent manner upon TC-A2317 treatment (Figure 5A and Supplementary Figure S4A). This finding indicates that the effects of TC-A2317 allow cells to overcome paclitaxel-induced mitotic arrest. Live-cell imaging revealed that most paclitaxel–treated cells persisted in mitotic arrest, and a subset underwent cell death (Figure 5B). By contrast, cells treated with paclitaxel plus TC-A2317 exited from mitotic arrest and then formed multinucleated cells (Figure 5B). To confirm the formation of multinucleated cells, nuclear morphology was visualized. Post-treatment of TC-A2317 in paclitaxel-induced arrested cells induced the multinucleation (Figure 5C and 5D). Similarly, co-treatment of paclitaxel and MLN-8054 induces multinucleation [20]. These observations demonstrate that TC-A2317–treated cells underwent mitotic slippage, and suggest that TC-A2317 perturbs cellular mechanisms involved in blocking mitotic progression even in the presence of improper mitotic spindles and interferes with maintenance of chromosomal stability.

### TC-A2317–treated cells fail to activate the SAC

**Figure 6:**
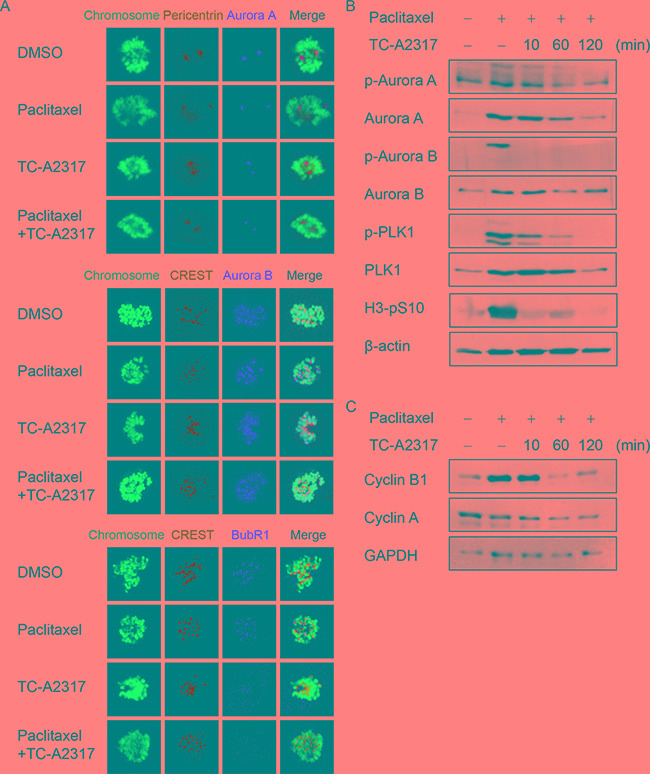
TC-A2317 inactivates paclitaxel-induced SAC **A.** A549 cells were treated with 50 nM paclitaxel for 16 hr, and then treated with 0.5 μM TC-A2317 for 30 min in the presence of 20 μM MG132. Localizations of Aurora A, Aurora B, and BubR1 were determined by staining with the indicated antibodies. **B-C.** A549 cells were treated with 50 nM paclitaxel for 16 hr, and then treated with 0.5 μM TC-A2317 for the indicated times. The level of each protein was determined by Western blotting.

To determine how TC-A2317 treatment allows cells to escape mitotic arrest, we performed immunofluorescence staining to monitor the localizations of mitotic kinases, including Aurora A, Aurora B, and BubR1, all of which regulate the SAC. Aurora A was localized to the centrosome, as determined by staining for pericentrin. In control cells, Aurora A and pericentrin were merged in prometaphase. The two proteins were also co-localized in cells treated with paclitaxel, TC-A2317, or paclitaxel plus TC-A2317 (Figure 6A and Supplementary Figure S5A). In all cells, Aurora B was localized to kinetochores, as determined by staining with CREST, an anti-centromere autoantibody from sera of patients with CREST syndrome (limited scleroderma) (Figure 6A and Supplementary Figure S5A). BubR1 was localized to kinetochores in prometaphase of control cells and paclitaxel–treated cells (Figure 6A), but not in those of cells treated with TC-A2317 or paclitaxel plus TC-A2317 (Figure 6A and Supplementary Figure S5A). This was consistent with a previous finding that alisertib reduces recruitment of Bub1 and BubR1 to the kinetochore in nocodazole–treated cells [21]. Together, these data demonstrate that TC-A2317 prevents full activation of SAC. As described in Figure 3B, the distance between two centrosomes is closer in TC-A2317-treated cells than control cells (Figure 6A and Supplementary Figure S5A) during mitosis, indicating that TC-A2317 treatment results in a defect in centrosome separation. In addition to determining the localization of SAC regulators, we monitored SAC activation by assessing the phosphorylation of each of the proteins involved (Figure 6B and Supplementary Figure S4B). Although paclitaxel increased the level of p-Aurora A, p-Aurora B, and p-PLK1, TC-A2317 decreased the levels of the corresponding phosphoproteins even in the presence of paclitaxel. Thus, TC-A2317 overrides the SAC induced by anti-mitotic agents. To confirm whether cells exited from paclitaxel-induced mitotic arrest, the levels of H3-pS10, cyclin B1 and cyclin A were examined. The high level of H3-pS10 and cyclin B1 induced by paclitaxel treatment dramatically decreased upon TC-A2317 treatment (Figure 6B and 6C). By contrast, the level of cyclin A was not changed (Figure 6C), suggesting that the enriched population with H3-pS10-negative 4N DNA was not G2 cells (Figure 5A). It indicates that the cells exited from mitosis reach the next phase of cell cycle. Overall, the data suggest that TC-A2317–treated cells lose the ability to ensure proper regulation of chromosomal stability.

### Aurora kinase A is a potential target for cancer therapy

**Figure 7:**
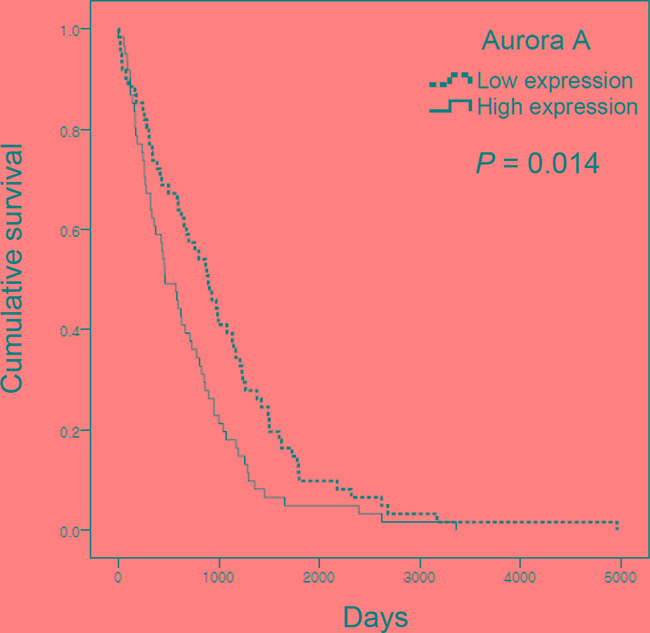
Aurora A expression is associated with low survival of lung adenocarcinoma cancer patients The mRNA expression data set was obtained from TCGA. Kaplan–Meier survival analysis was performed on 122 dead patients. Aurora A expression was defined as high (above median) or low (below median). *P*-values were based on the log-rank test.

Aurora A is widely overexpressed in many cancers [8], suggesting that inhibition of the Aurora kinase A is a potential target for cancer therapy. We downloaded the *AURKA* mRNA levels from TCGA dataset and performed Kaplan-Meier analysis. Kaplan–Meier curves demonstrated that lung cancer patients with high level of *AURKA* had significantly poorer survival (Figure 7). Thus, Aurora A expression is suggested as a strong predictive value for survival of lung cancer patients.

## DISCUSSION

In this study, we showed that the Aurora kinase A inhibitor TC-A2317 induces abnormal centrosome formation and aberrant mitotic spindles. Even in the presence of mitotic malfunction, TC-A2317-treated cells undergo mitotic slippage. The resultant chromosome mis-segregation leads to inhibition of lung cancer cell proliferation via induction of apoptosis, autophagy, or senescence. In addition, TC-A2317 sensitizes cells to paclitaxel-induced mitotic catastrophe such as multinucleation via an inactivation of SAC.

Inhibition of Aurora kinase A activity by different inhibitors leads to similar phenotypes. ENMD-2076 inhibits proliferation of breast, colon and ovarian cancer, and multiple myeloma *in vivo* and *in vitro*. EMND-2076–treated cells showed polyploidy and apoptotic cells [22, 23]. MK-5108 (VX-689) inhibits proliferation of breast, colon, and non-small cell lung cancer, leukemia, nasopharyngeal carcinoma, uterine leiomyosarcoma, and ovarian cancer stem cells *in vivo* and *in vitro*. The duration of G2 is prolonged by MK-5108 treatment, as also observed in cells treated with siRNA targeting Aurora A [24]. MK-5108 treatment also prolongs mitosis [25, 26] and increases the proportion of H3-pS10–positive mitotic cells [25, 27–30]. MK-5108–treated cells undergo apoptosis [28, 30]. MLN-8054 inhibits proliferation of colon, prostate, and thyroid cancer, melanoma and neuroblastoma *in vivo and in vitro*. MLN-8054 treatment results in prolonged mitosis [31], abnormal formation of centrosomes and mitotic spindles [31], aneuploidy including multinucleation, micronucleation, and polyploidy [21, 31–36], apoptosis [21, 32, 33, 35], and senescence [37]. The most extensively studied Aurora kinase A inhibitor is an MLN-8237 (alisertib) [38]. Alisertib inhibits proliferation of bladder, breast, cervical, colorectal, esophageal, gastric, lung, pancreatic, and thyroid cancer as well as glioblastoma, leukemia, lymphoma, multiple myeloma, osteosarcoma, tongue squamous cell carcinoma, and uterine leiomyosarcoma. In addition, alisertib treatment induces delayed mitotic entry [39], prolonged mitotic duration [26, 39], defects in mitotic spindle formation [39–42] and cytokinesis [43, 44], multi-centrosomes [39], polyploidy [26, 39, 40, 43–52], DNA damage [36], apoptosis [19, 44–49, 52–57], autophagy [46, 47, 53–55], and senescence [19, 36, 40, 52, 56].

The novel Aurora kinase A inhibitor TC-A2317 also causes defects in the mitotic spindle, abnormal centrosome, prolonged mitotic duration, aneuploidy, apoptosis, autophagy, and senescence. In comparison with previous studies, distinctive findings of this study include the observations of mitosis portion and instability of centrosomes and microtubules. MK-5108–treated cells are H3-pS10–positive [25, 27–30], indicating that cells are arrested at mitosis. However, as observed in our study, MLN-8237–treated cells contain low levels of H3-pS10 *in vitro* [43]. TC-A2317 treatment for 48 and 72 hr significantly decreased it, indicating that the cells were not ultimately arrested at mitosis (Figure 2B). Xenograft tumors isolated from mice orally treated with alisertib contain the highest level of H3-pS10 at 8–12 hr, but lower levels thereafter [50]. These observations suggest that Aurora kinase A inhibitors initially prolong mitotic progression and arrest cells in mitosis, but that the accumulated chromosomal instability eventually overrides the SAC, resulting in permanent cell cycle arrest (i.e., senescence) with polyploidy or apoptosis. Next, the chromosomal instability induced by Aurora kinase A inhibition might be due to defects in centrosome and mitotic spindle formation. The second difference between the results of this study and previous reports involves centrosome number. Brief treatment (5 hr) with MLN-8054 leads to the formation of monocentrosome and multipolar spindles. By contrast, longer treatment (>24 hr) results in centrosome amplification [31]. Treatment with alisertib for 24 hr induces formation of monopolar spindles in glioblastoma stem cells, but multipolar spindles in differentiated glioma cells [40]. By contrast, in our study, TC-A2317 induced a defect in centrosome separation during mitosis (Figure 3B, Figure 6A and Supplementary Figure S5A), and finally a formation of monocentrosome in interphase (Figure 3A). Third, brief treatment (4 hr) with a high dose of alisertib treatment dramatically increases the proportion of cells with no mitotic spindles [39]. Alisertib treatment also causes loss of inter-microtubule bridges by disrupting the TACC3/ch-TOG/clathrin complex and prevents cold-stable K-fiber attachment to the kinetochore [42, 58]. Our study also demonstrates that TC-A2317–treated cells have unstable mitotic spindles. However, acute treatment with alisertib (15 min) results in microtubule hyperstabilization and subsequent spindle pole fragmentation [41]. Thus, these differences in centrosomes and mitotic spindles might be dependent on both the identity of the specific drug used and the incubation time. Moreover, although TC-A2317-mediated SAC inactivation leads to mitotic slippage, mitotic duration is not shortened; instead, the prolonged mitotic duration might be due to abnormal centrosome separation and microtuble destabilization.

The significant reduction in cell proliferation resulting from treatment with Aurora kinase A inhibitor can be explained by induction of apoptosis, autophagic cell death, or senescence, all of which can be regulated by p53. In fact, Aurora A regulates the level and activity of p53 by phosphorylating residue Ser315, thereby destabilizing the protein [59], as well as Ser215, thereby diminishing DNA-binding activity [60]. Consistent with this finding, treatment with Aurora kinase A inhibitors such as MK-5108, MK-8054, or alisertib induces upregulation of p53 [21, 26, 37, 55, 61]. However, when p53 is deleted in breast cancer, alisertib induces senescence rather than apoptosis [56]. In HCT116 p53^-/-^ cells, MK-5108 treatment prolongs mitosis and generates more polyploidy after mitotic slippage [26]. In addition, MK-8745, a specific Aurora kinase A inhibitor, also causes apoptosis in HCT116 p53^+/+^ cells, but induces polyploidy in HCT116 p53^-/-^ cells [62]. Overall, these findings demonstrate that p53 is required for induction of apoptosis by Aurora kinase A inhibition; in the absence of p53, the same treatment aggravates polyploidy and induces senescence. Consistent with the previous findings, our study also demonstrated that p53-null NCI-H1299 cells dramatically underwent senescence than p53-wild type A549 and A427 cells. However, NCI-H1299 cells also underwent significant apoptosis even in the absence of p53, suggesting that other apoptosis-inducing factors are involved. The senescence induced by Aurora kinase A inhibition might be mediated by cyclin-dependent kinase inhibitor p21. In this study, except for A427 cells, A549 and NCI-H1299 cells treated with TC-A2317 upregulated p21, as previously observed in MLN-8054–treated HCT116 cells [37] and MLN-8237–treated HT29 and Caco-2 cells [54]. However, although p53 was upregulated in TC-A2317-treated A427 cells, p21 was not changed, supporting that TC-A2317 did not induce senescence in A427 cells. The different phenomenon between A549 and A427 cells could be addressed by K-ras mutation. While A549 cells harbor K-ras^G12S^, A427 cells contain K-ras^G12D^ which suppresses senescence [63]. Overall, the p53 or other factor can induce apoptosis, and p21 can induce senescence in TC-A2317–treated cells.

The SAC is activated when mitotic spindles are not correctly attached to kinetochores. SAC activation at unattached kinetochores halts the onset of anaphase to prevent separation of sister chromatids until the chromosomes are properly attached [1]. Several anti-mitotic agents that interfere with microtubule dynamics, including paclitaxel, vincristine, and vinblastine, activate SAC, ultimately inducing mitotic arrest and mitotic catastrophe. Therefore, anti-mitotic agents have been utilized as conventional anti-cancer drugs. Because pharmacological inhibition of Aurora kinase A overrides SAC-mediated mitotic arrest and aggravates chromosomal instability, the combination of anti-mitotic agents and Aurora kinase A inhibitor enhances chemosensitivity. Aurora kinase A inhibition by MK-5108 and alisertib increases the anti-tumor activity of docetaxel, paclitaxel, and vincristine [27, 48, 51, 64–66], whereas Aurora A overexpression increases resistance to taxol [67]. Overall, targeting the mitotic functions of Aurora A using pharmacological inhibitors increases the anti-cancer effect of anti-mitotic agents.

## MATERIALS AND METHODS

### Cell culture

A549 (p53-wild type, p16-null) and A427 (p53-wild type, p16-null) non-small cell lung cancer cells were obtained from the American Type Culture Collection (ATCC, Lot No. 58314291 and 58696830, respectively) and maintained in Dulbecco's modified Eagle's medium (DMEM, Welgene Inc.). NCI-H1299 (p53-null, p16-deficient) cells were obtained from the Korean Cell Line Bank (KCLB) and maintained in Roswell Park Memorial Institute (RPMI) 1640. All media were supplemented with 10% fetal bovine serum (FBS), 100 U/ml penicillin G sodium, 100 μg/ml streptomycin sulfate, and 0.25 μg/ml amphotericin B. Cells were incubated at 37 °C in 5% CO_2_ incubator.

### Drug treatment

Cells were treated with 0.5 or 1 μM TC-A2317 (Tocris, 4066), 0.5 μM alisertib (Selleckchem, S1133) or 50 nM paclitaxel (EMD Millipore, 580555) for indicated times. All drugs are dissoved in DMSO as a vehicle. The final concentration of vehicle in culture medium was 0.1%.

### Cell viability assay

Cells (7.5×10^3^ cells per well) were plated in a 96-well plate and then treated with TC-A2317 for 24 hr. MTT (3-(4,5-dimethylthiazol-2-yl)-2,5-diphenyltetrazolium bromide) was added to each well, and the plate was incubated at 37°C for 4 hr to allow formation of MTT formazan crystals. After the culture medium was removed, the formazan crystals was dissolved using dimethyl sulfoxide. The absorbance was measured with a test wavelength of 570 nm and a reference wavelength of 650 nm [68].

### Clonogenic assay

Cells (5×10^2^ cells per 60 mm dish) were plated and then incubated for 14 days. After removal of the medium, cells were rinsed with phosphate-buffered saline (PBS), fixed in acetic acid:methanol (1:7, vol/vol) at room temperature for 5 min, and then stained with staining solution (0.5% crystal violet in 25% methanol). Colonies were counted on triplicate dishes, and independent experiments were repeatedly done [68].

### Cell cycle analysis

Cells were suspended in PBS and then 100% ethanol was added to be the final concentration of 70% ethanol while gently vortexing. The fixed cells were permeabilized with 0.25% Triton X-100 in PBS on ice for 15 min. The cells were incubated with anti-H3-pS10 (Histone H3 phosphorylation at S10, Upstate, 06-570) antibody for 2 hr, and then incubated with FITC-conjugated goat anti-rabbit IgG (Jackson ImmunoResearch Laboratories Inc., 111-095-144) at room temperature in the dark for 1 hr. Cells were incubated with DNase-free RNase A at 37 °C for 30 min and then with propidium iodide (PI) at 37 °C in the dark for another 30 min. The cell cycle phase and H3-pS10-positive cells were determined by flow cytometry and analyzed using C6 software. The percentage of each cycle phase was analyzed using Modfit software [68].

### Immunofluorescence staining

Cells grown on coverslips were fixed with 3% paraformaldehyde solution at room temperature for 10 min and then permeabilized with 0.5% Triton X-100 at room temperature for 5 min. The cells were incubated with antibody against Aurora A (BD Biosciences, 610938), Aurora B (Santa Cruz, sc-25426), BubR1 (BD Biosciences, 612503), Pericentrin (Abcam, 28144) or CREST (ImmunoVision, HCT-0100) at 37 °C for 20 min and then incubated with corresponding secondary antibody at 37 °C for 20 min. For the staining with α-tubulin (Abcam, 18251) and pericentrin antibodies, the cells were fixed with cold methanol at -20 °C for 20 min and then rehydrated in PBS three times. The cells were post-fixed with paraformaldehyde and permeabilized as described above. The nuclei were counterstained with Hoechst 33342. After a final wash with PBS, coverslips were mounted with antifade solution containing para-phenylenediamine and glycerol in PBS. The staining was determined using laser-scanning confocal microsope (LSM700, Carl Zeiss). Images are acquired using ZEN software (Carl Zeiss) [68].

### Microtubule depolymerization assay

Cells grown on coverslips were cultured in the presence of 2 mM thymidine for 19 hours and then released to grow for 10 hr. Cells were then treated with 2 mM thymidine for another 15 hours, causing cells to arrest at the G1/S boundary. The thymidine was washed off with PBS, and the arrested cells were allowed to progress to mitosis for 10 hr. To block the anaphase transition, 20 μM MG132 was added for 1 hr. Subsequently, the cells were incubated with TC-A2317 in cold media on ice for 5 min in the absence of MG132. Cells were stained with α-tubulin antibody, and metaphase cells with depolymerized mitotic spindles were counted [41].

### Time-lapse microscopy

TSiN-H2B-RFP lentiviral construct was kindly gifted by Dr. P. J. Galardy (Mayo Clinic). Lentivirus was prepared by transfection of HEK293T cells with TSiN-H2B-RFP lentiviral plasmid, psPAX2 packaging plasmid, and pMD2.G envelope plasmid. A549 cells were infected with lentivirus encoding H2B-RFP in the presence of 8 μg/ml polybrene. Time-lapse imaging was acquired using a Cell Observer (Cell Observer Living Cells, Carl Zeiss) equipped with a camera and Axiovision (Carl Zeiss). Frames were recorded every 5 min. Cell morphology was visualized on a phase-contrast microscope, and RFP was detected by fluorescence [68].

### Western blotting

Cells were lysed using NETN buffer (100 mM NaCl, 1 mM EDTA, 20 mM Tris-HCl pH 8.0, 0.5% Nonidet P-40, 50 mM β-glycerophosphate, 10 mM NaF and 1 mM Na_3_VO_4_) with protease inhibitor (Millipore, 535140) on ice for 10 min. After centrifugation at 12,000 ×g for 5 min, the supernatant was saved as a crude cell extract. The crude cell extracts were boiled in the Laemmli buffer and then loaded onto a SDS-polyacrylamide gel [68]. The antibodies used for Western blotting are as follows: PARP-1 (Santa Cruz Biotechnology, sc-7150), p62/SQSTM1 (Cell signaling, 5114), LC3 (MBL International, PM036), Cyclin A (Santa Cruz Biotechnology, sc-751), Cyclin B1 (Santa Cruz Biotechnology, sc-752), Cyclin D1 (Santa Cruz Biotechnology, sc-753), p53 (Santa Cruz Biotechnology, sc-126), p21 (EMD Millipore, OP64), Aurora A-pT288 (Cell Signaling, 3079), Aurora A (Cell Signaling, 4718), Aurora A-pT288/Aurora B-pT232/Aurora C-pT198 (Cell Signaling, 2914), Aurora B (Cell Signaling, 3094), PLK1-pT210 (Santa Cruz Biotechnology, sc-135706), PLK1 (Cell Signaling, 4513), H3-pS10 (Upstate, 06-570), β-actin (Cell Signaling, 4970), and GAPDH (Santa Cruz Biotechnology, sc-25778).

### Senescence-associated β-galactosidase staining

The cells were washed with PBS, then fixed and stained at pH 6.0 using a senescence β-galactosidases (SA-β-gal) staining kit (Cell Signaling, 9860) according to the manufacturer's instructions [69].

### Statistical analysis

All experiments were done three times. Each value was expressed as means ± standard deviations. Student's t-test was used for statistical analysis and statistical difference was considered significant when *P* < 0.05.

### Association analysis of aurora kinase a expression with patient survival

Expression data were downloaded from The Cancer Genome Atlas (TCGA) (https://tcga-data.nci.nih.gov). *AURKA* transcript level in lung adenocarcinoma patients was determined by RNA-Seq V2 and processed using the SUBIO platform (version 1.19). For each sample, Aurora A expression was defined as high (above median) or low (below median). The association of transcript level with patient survival was visualized using Kaplan–Meier curves, and significance of differences was assessed by log-rank test using SPSS (version 12.0).

## SUPPLEMENTARY MATERIALS FIGURES



## Abbreviations

SAC: Spindle assembly checkpoint
