# Supplementary material for: The Aurora kinase A inhibitor TC-A2317 disrupts mitotic progression and inhibits cancer cell proliferation
Source: Oncotarget. 2016 Oct 4;7(51):84718–35. doi: 10.18632/oncotarget.12448 (PMC5356694; doi:10.18632/oncotarget.12448)
Supplement: Supplementary file 1 [file oncotarget-07-84718-s001.pdf]

The Aurora kinase A inhibitor TC-A2317 disrupts mitotic progression and inhibits cancer cell proliferation

SUPPLEMENTARY FIGURES

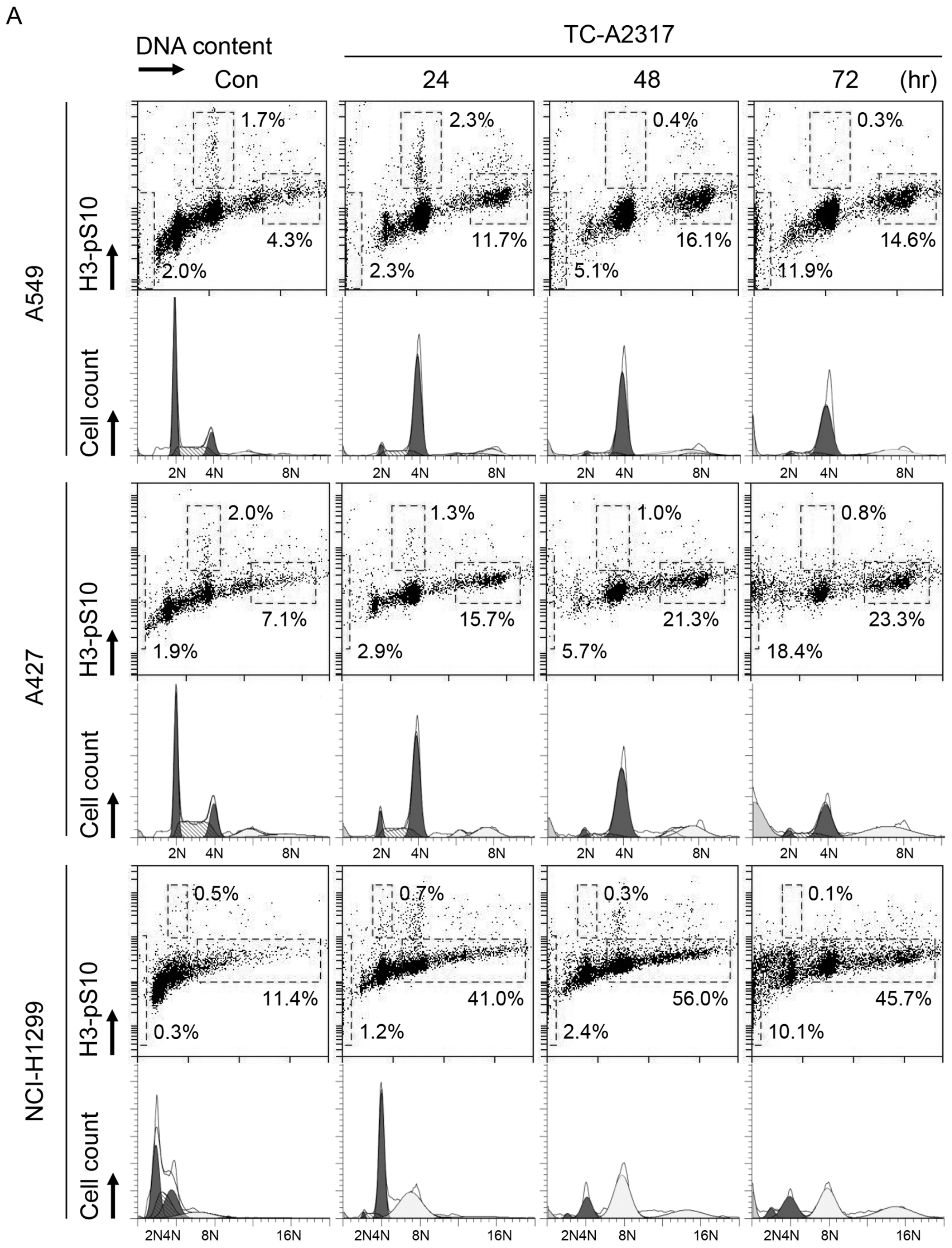

Supplementary Figure S1: TC-A2317 induces the abnormal progression of cell cycle. DNA content and mitotic fraction were analyzed by flow cytometry by staining cells with PI and anti–H3-pS10. Con (Control) means cells treated with vehicle (DMSO) for 72 hr. **A.** A549, A427 and NCI-H1299 cells were treated with 1 µM TC-A2317 for the indicated times. (*Continued*)

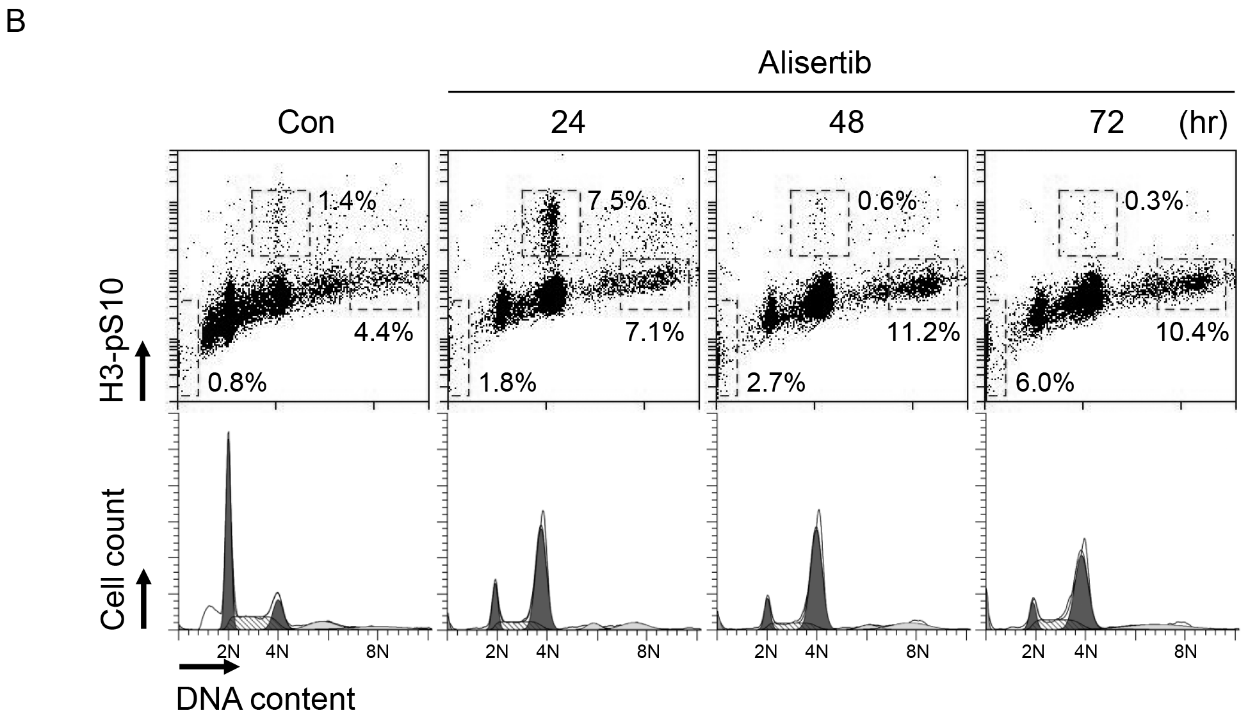

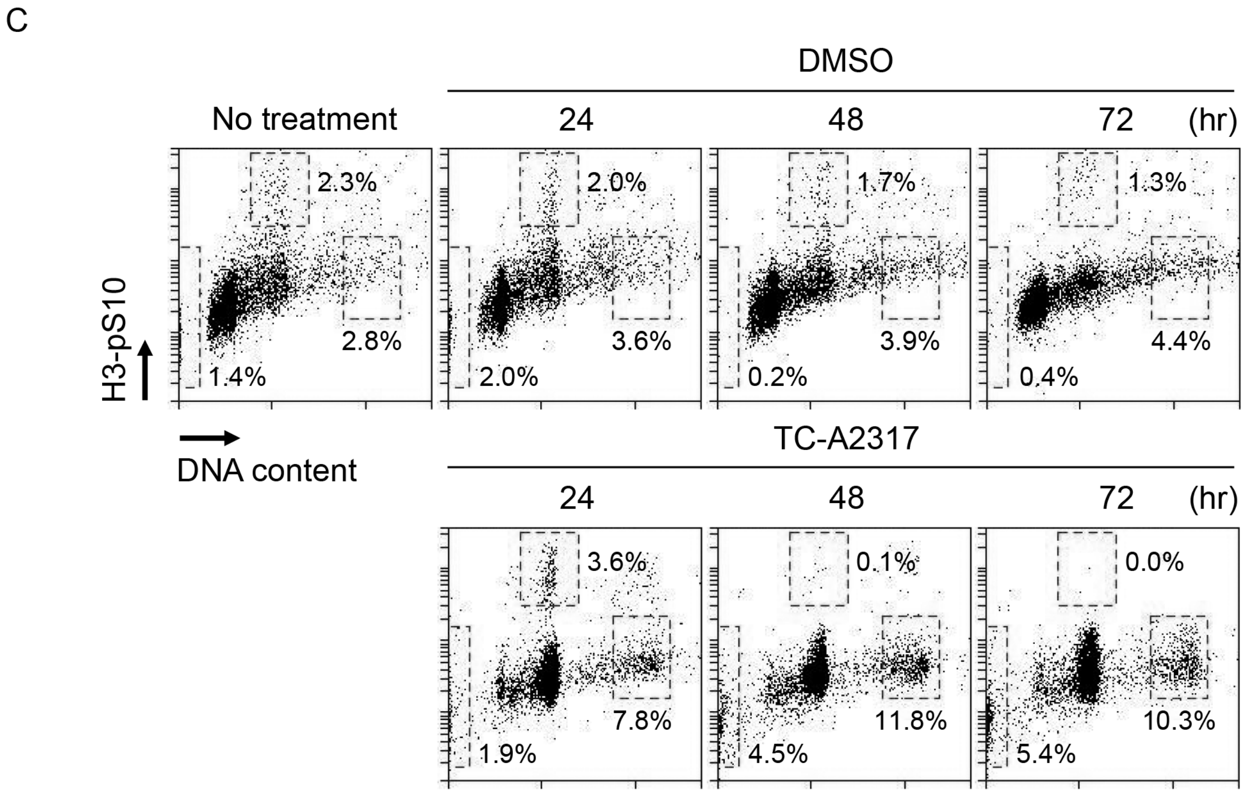

Supplementary Figure S1: (*Continued*) TC-A2317 induces the abnormal progression of cell cycle. B. A549 cells were treated with 0.5 μM alisertib for the indicated times. **C.** A549 cells were treated with 0.1% DMSO or 1 μM TC-A2317 for the indicated times.

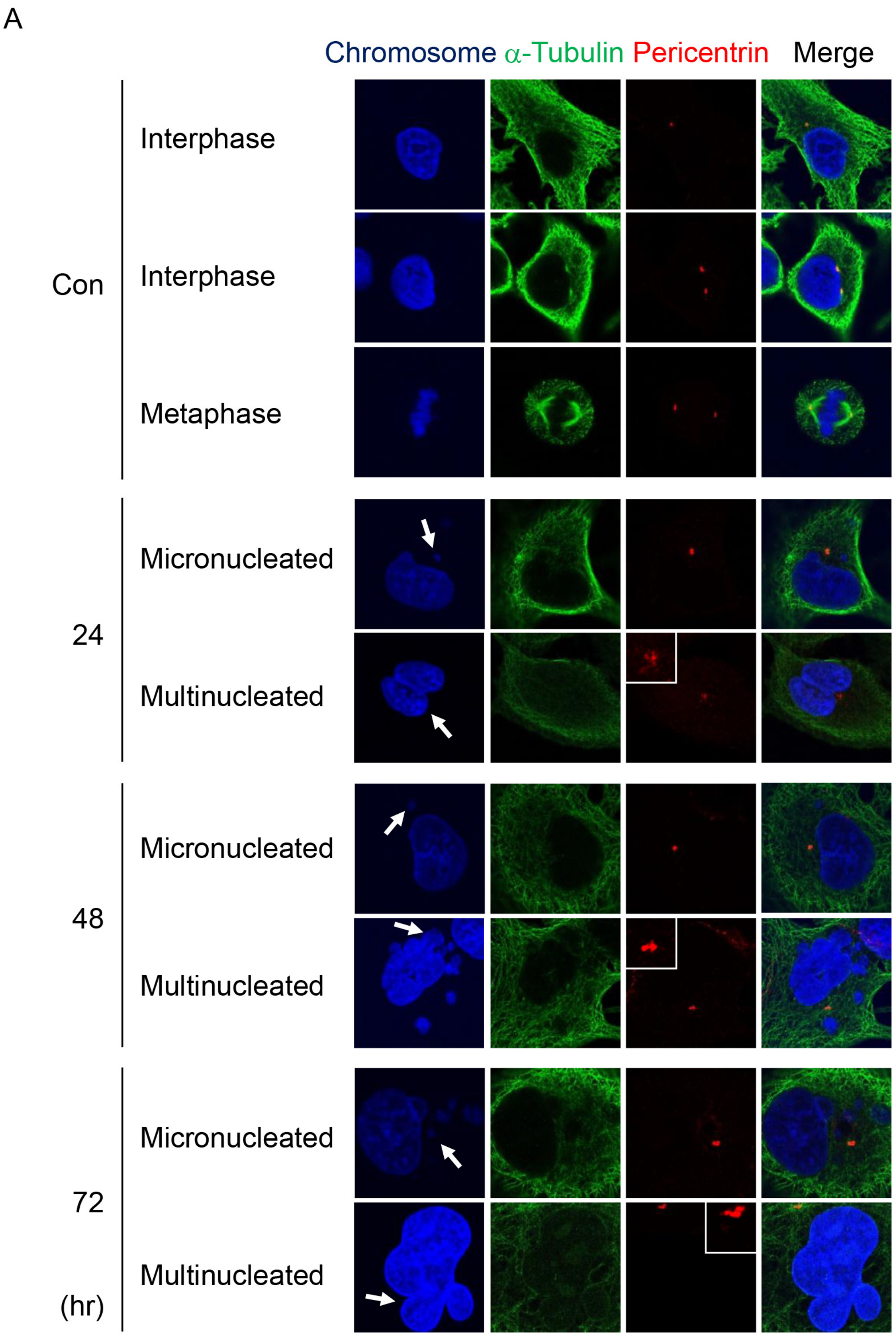

Supplementary Figure S2: Alisertib induces the formation of aneuploidy and abnormal centrosome. A. A549 cells were treated with 0.5 μM alisertib for the indicated times, and then subjected to immunofluorescence staining with antibodies against α-tubulin and pericentrin. Con (Control) means cells treated with vehicle (DMSO) for 24 hr.

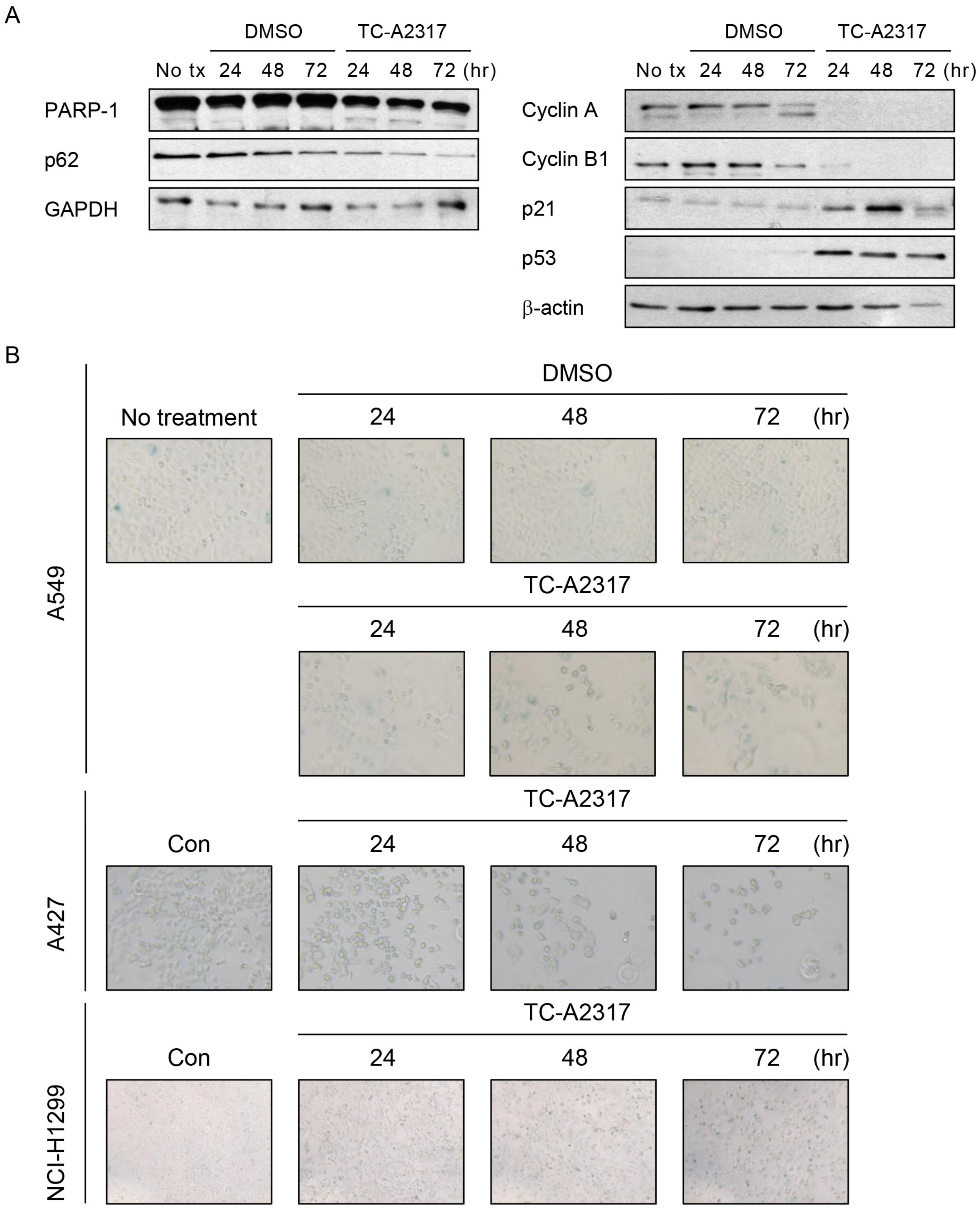

Supplementary Figure S3: TC-A2317 induces senescence in A549 and NCI-H1299, but not in A427 cells. A. A549 cells were treated with 0.1% DMSO or 1 μM TC-A2317 for the indicated times. Expression level of each protein was evaluated by Western blotting. **B.** Senescence-associated b-galactosidase (SA-b-Gal) assay was performed. A549, A427 and NCI-H1299 cells were treated with 0.1% DMSO or 1 μM TC-A2317 for the indicated times. Con (Control) means cells treated with vehicle (DMSO) for 72 hr.

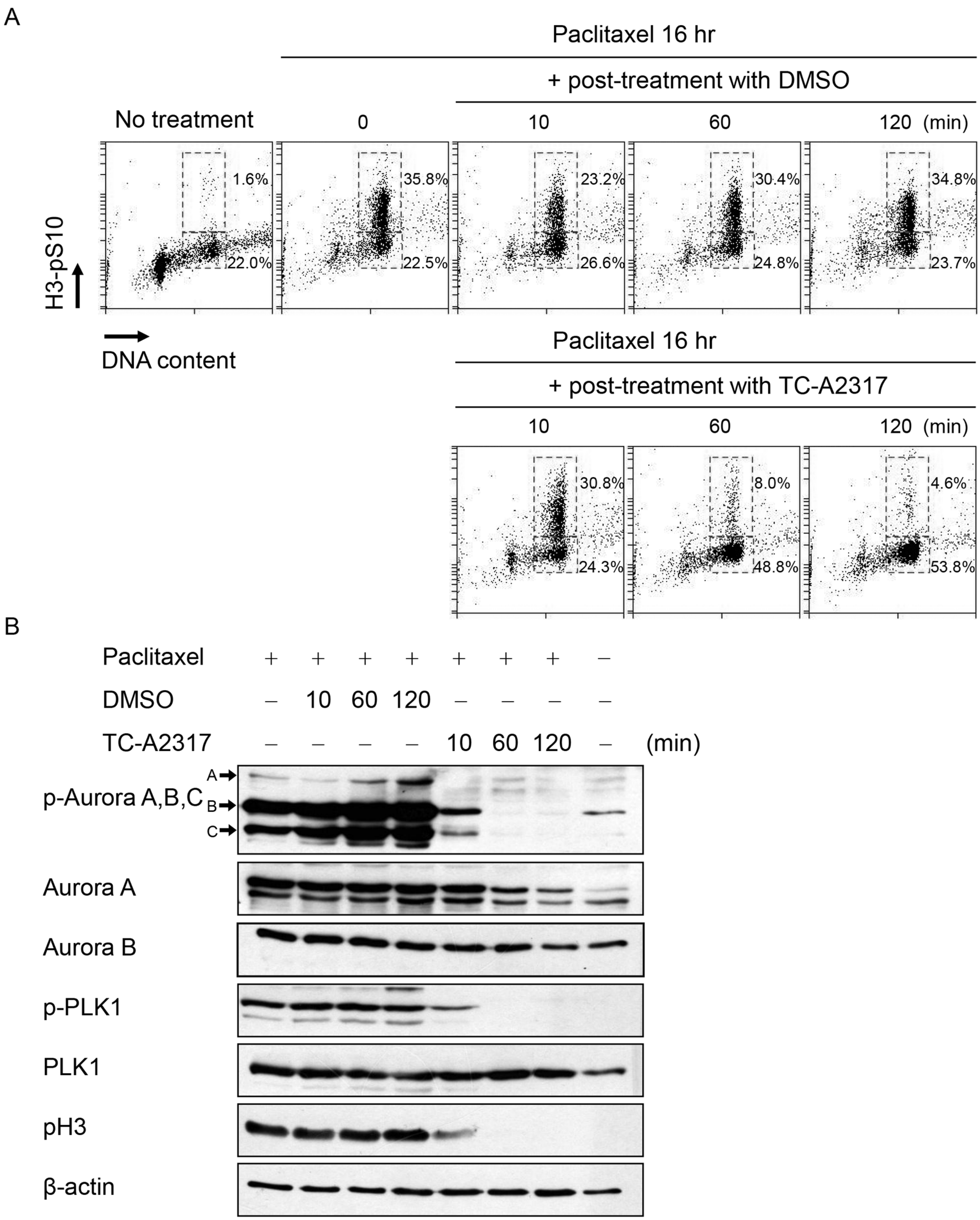

Supplementary Figure S4: DMSO used as a vehicle does not affect mitotic slippage. A-B. A549 cells were treated with 50 nM paclitaxel for 16 hr, and then treated with 0.05% DMSO or 0.5 µM TC-A2317 for the indicated times. **(A)** DNA content and H3-pS10 level were determined by flow cytometry. **(B)** The level of each protein was determined by Western blotting.

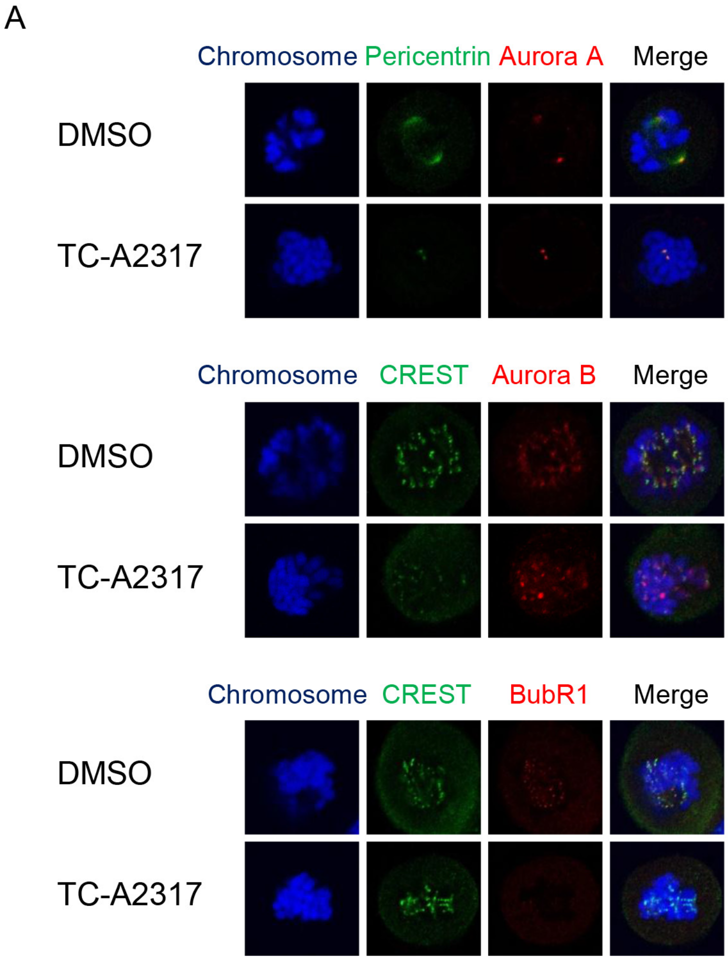

Supplementary Figure S5: TC-A2317 inactivates SAC at prometaphase. A. A549 cells were treated with 1 μM TC-A2317 for 1 hr in the absence of MG132. Localizations of Aurora A, Aurora B, and BubR1 were determined by staining with the indicated antibodies.
